# Identification of a novel mechanism for regulation of the early autophagy machinery assembly by PKA

**DOI:** 10.1080/27694127.2025.2503226

**Published:** 2025-05-11

**Authors:** Miranda Bueno-Arribas, Olivier Vincent

**Affiliations:** a Instituto de Investigaciones Biomédicas Sols-Morreale CSIC-UAM, Madrid, Spain; b Institut Necker Enfants-Malades, Team 8, INSERM UMR-1151, CNRS UMR-8253, Université Paris Cité, Paris, France

**Keywords:** Autophagy, ATG17, ATG12, PKA, reverse two-hybrid, protein complex structure prediction, phosphoregulation

## Abstract

The E3-like complex Atg12-Atg5-Atg16, which promotes Atg8 lipidation, is recruited to the autophagosomal membrane through the interaction of Atg16 with the PROPPIN/WIPI protein Atg21, as well as by the binding of Atg12 to Atg17, the scaffold protein of the Atg1 kinase complex in yeast. In order to gain insights into the molecular basis of Atg12-Atg17 interaction, we performed reverse two-hybrid screens to identify key-binding residues in both proteins and, based on these data, model the structure of this protein complex. Strikingly, we found that the Atg17 binding site in Atg12 overlaps with a PKA phosphorylation site and that PKA phosphorylation of Atg12 prevents Atg17 binding, revealing a new regulatory mechanism by which PKA regulates the assembly of the autophagy machinery.

During macroautophagy/autophagy, a key step in autophagosome formation is the conjugation of Atg8 to phosphatidylethanolamine in the autophagosomal membrane by the E3-like complex Atg12-Atg5-Atg16. Initially, it was shown that recruitment of this complex to the phagophore assembly site (PAS) is mediated by the interaction of Atg16 with the phosphoinositide-binding protein Atg21. More recently, however, an additional mechanism for this E3-like complex recruitment to the PAS was identified and involves the binding of Atg12 to Atg17, the scaffold protein of the Atg1 kinase complex, which is key in initiating autophagosome formation.

In our recent work [1], we used the reverse two-hybrid system to investigate the molecular basis of Atg12-Atg17 complex formation. This method allows the identification of residues critical for protein–protein interaction by selecting randomly generated missense mutations that specifically prevent the binding between the two proteins. Results from two-hybrid analysis were further validated by pull-down assays.

Our results show that the Atg12 binding site is located on the convex side of the Atg17 crescent structure, in a region that has not been previously implicated in the interaction of this scaffold protein with other components of the Atg1 kinase complex. On the other hand, the Atg17 binding site in Atg12 resides on a short alpha-helix segment in the intrinsically disordered N-terminal region of this protein. Based on these results, we modeled the structure of the Atg12-Atg17 complex using the docking program HADDOCK. The resulting model shows that the alpha helix in the N-terminal region of Atg12 could form a four-helix bundle with the three helices of Atg17, similar to the mechanism described for Atg31 binding to Atg17. However, binding of Atg12 and Atg31 to opposite sides of Atg17 suggests that these proteins do not compete for access to the scaffold protein. Our model differs in part from a model previously generated in a large-scale analysis with RoseTTAFold/AlphaFold that places the Atg12 binding site on the opposite side of the Atg17 crescent structure, highlighting the need to validate models generated by artificial intelligence with experimental data.

To specifically address the physiological relevance of the association between the Atg1 kinase complex and the Atg8 conjugation machinery mediated by Atg12-Atg17 binding, we studied the effect of loss-of-binding mutations in Atg12 (R53G, L54S, S55P, L57P) and Atg17 (M91V, L137S, L141P) on autophagic flux. Consistent with previous work, we found these mutations result in a partial inhibition of autophagy, especially when the second recruitment mechanism of the Atg12-Atg5-Atg16 E3-like complex, mediated by Atg21, is blocked. However, in contrast to what has been previously reported, inactivation of both recruitment mechanisms does not completely block autophagy, supporting the notion of the existence of at least one alternative mechanism, possibly mediated by the binding of Atg16 and/or Atg5 to the autophagosomal membrane.

Strikingly, we found that the Atg17 binding site in Atg12 overlaps with a protein kinase A (PKA) phosphorylation consensus sequence, raising the possibility that this interaction is regulated through phosphorylation by this protein kinase. We confirmed that the Ser55 residue in Atg12, within the Atg17 binding site, is phosphorylated by PKA *in vivo*, and that phosphorylation of this residue in Atg12 prevents the interaction with Atg17. Previous studies have shown that PKA inhibits autophagy by phosphorylating Atg1 and Atg13, and that PKA inactivation is sufficient to induce autophagy in nutrient-rich conditions. Phosphorylation of Atg13 by PKA, like that of Atg12, prevents Atg17 binding, and PKA phosphorylation of Atg1 also prevents its recruitment to the PAS. To assess the contribution of these different regulatory mechanisms to PKA-mediated inhibition of autophagy, we tested whether simultaneous inactivation of the different phosphorylation sites in Atg1, Atg13, and Atg12 activates autophagy in nutrient-rich conditions. However, we only detected a slight increase in autophagic flux under these conditions, suggesting the existence of additional regulatory mechanisms by which this signaling pathway controls the autophagy machinery.

Both TORC1 (target of rapamycin complex 1) and PKA inhibit the formation of the autophagy initiation complex by phosphorylating Atg13 and Atg1 (Figure 1). Furthermore, inhibition of either TORC1 or PKA induces autophagy, suggesting that each of these pathways alone is not sufficient to block the formation of the initiation complex. This mechanism would ensure that autophagy is induced in response to stimuli that inhibit either of these two pathways. However, the physiological relevance of this mechanism for PKA is unknown, and it is possible that the function of this signaling pathway is to modulate the magnitude of the response depending on the availability of carbon sources.

**Figure 1.**
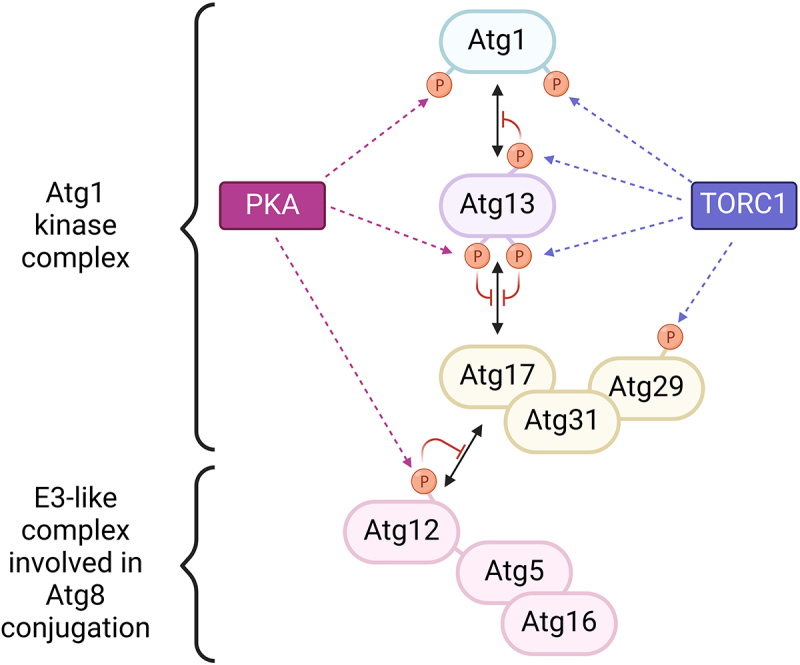
Model for regulation of the Atg1 kinase complex by TORC1 and PKA. Phosphorylation of Atg13 by both kinases inhibits its binding to Atg17. Furthermore, phosphorylation of Atg13 by TORC1 also prevents its interaction with Atg1. PKA and TORC1 also converge on the phosphorylation of Atg1. Phosphorylation of Atg1 by PKA prevents its association with the PAS but does not appear to alter Atg1-Atg13 binding, while TORC1 phosphorylation of Atg1 has not been characterized yet. TORC1 further phosphorylates Atg29 and PKA phosphorylates Atg12, which prevents Atg17 binding and thereby the association of the E3-like complex involved in Atg8 conjugation with the Atg1 kinase complex.

Our results show that PKA also prevents the association of the autophagy initiation complex with the E3-like Atg12-Atg5-Atg16 complex of the conjugation machinery (Figure 1). This interaction has been reported to not only stimulate Atg8 lipidation but also promote PAS scaffold assembly. Therefore, these results support the idea that PKA inhibits PAS assembly at different levels, involving the formation of the Atg1 kinase complex and its association with the Atg8 conjugation machinery.

Interestingly, the association between the Atg1 kinase complex and the E3-like complex of the Atg8 conjugation machinery has also been described in mammals, although in this case, the Atg17 functional counterpart, FIP200, interacts with another subunit of this E3-like complex, ATG16L1. PKA also phosphorylates ATG16L1 on residues in proximity to the FIP200 binding sites, highlighting that this regulatory mechanism may also occur in mammals.

## Abbreviations

PAS: phagophore assembly site
PKA: protein kinase A
TORC1: target of rapamycin complex 1
